# Global health landscape challenges triggered by COVID-19

**DOI:** 10.1186/s41232-020-00144-5

**Published:** 2020-09-15

**Authors:** Hiroki Nakatani, Kei Katsuno, Hayato Urabe

**Affiliations:** 1 Global Research Institute, Keio University, Room5N6, Center for Integrated Medical Research, School of Medicine, Keio University, 35 Shinanomachi, Shinjuku-ku, Tokyo, 161-8582 Japan; 2 Global Health Innovative Technology (GHIT) Fund, Tokyo, Japan; 3 National Center for Global Health and Medicine, Tokyo, Japan; 4 Investment Strategy, Business Development, GHIT Fund, Tokyo, Japan; 5 Investment Strategy, Portfolio Development & Innovations, GHIT Fund, Tokyo, Japan

**Keywords:** Global health, COVID-19, Access to medicine, Research and development (R&D)

## Abstract

The COVID-19 pandemic highlighted the vulnerability of every aspect of the globalized world, including R&D. Potentially critical R&D areas have been neglected because of the lack of market-driven incentives. However, new initiatives are emerging to address the present crisis of COVID-19 and possibly future similar incidents that will threaten humanity. In this paper, the global health landscape of R&D is discussed in terms of research focus and funding, illustrating under-funding in communicable diseases with the exception of three major infections: HIV/AIDS, tuberculosis, and malaria. The initiatives triggered by the COVID-19 pandemic and the novel emphasis on “access” are discussed. Finally, the authors propose a new funding model to address R&D in the case of market failure, by forming alliance between government, industry, and international philanthropic organization (GHIT model), and define clear strategy of enhancing access as the way forward.

## Introduction

The UN Secretary-General’s high-level panel on Ebola outbreak in 2014–15 submitted a report [1] to the UN General Assembly on 9 February 2016. It starts with the following line, “When 2-year-old Emile Ouamouno from Guinea contracted Ebola and died on 28 December 2013, little did anyone realize that it would set off a chain of events that would lead to the deaths of more than 11,000 people, create worldwide fear, and require the mobilization of a multibillion-dollar global response.” The history is repeated on a much larger scale in the case of coronavirus disease 2019 (COVID-19) caused by a novel coronavirus now named SARS-CoV-2. On 31 December 2019, the WHO Country Office in China was informed of cases of pneumonia of unknown etiology detected in Wuhan City, Hubei Province of China [2]. On this New Year’s Eve, no one imagined that year 2020 will be plagued by COVID-19, which has infected more than 24 million people and claimed almost 840,000 lives as of 29 August 2020 affecting almost all countries. However, its impact on health, society, and economy evolves over time. The figures will grow, since after devastating cities in China, Europe, and the USA, the pandemic is now moving to Latin American, Russia, and then possibly Africa. This is an expected consequence of the globalized world, in which goods and people are moving in unprecedented scale and speed. The world has seen outbreaks that endangered the globe nearly every 5 years after the turn of the century: the SARS (severe acute respiratory syndrome) outbreak in Asia in 2003, H1N1 (influenza virus A/H1N1) pandemic in 2009, Ebola outbreak in West Africa in 2001, and COVID-19 pandemic in 2020. The previous three infections were finally controlled with resulting changes in global health architecture. SARS led the world to overhaul the quarantine system and establish the International Health Regulations (IHR) [3] that addresses the challenges of any health risk regardless of entity, including new infections and consequences of significant natural disasters. The IHR emphasizes timely sharing of information and strengthening of the national capacity of reporting and response. The H1NI pandemic resulted in the agreement of the Pandemic Influenza Preparedness (PIP) Framework [4] that was designed to increase access to vaccines for developing countries in exchange for smooth provision of specimens for vaccine development. Ebola outbreak stimulated the creation of the WHO Health Emergencies Programme, which is more operational than previous WHO works and involves mainly data collection and various standard setting. Also, the Coalition for Epidemic Preparedness Innovations (CEPI) [5], a new vaccine R&D mechanism for emerging infections, was established with the same funding model pioneered by Global Health Innovative Technology (GHIT) Fund [6], the first international R&D fund initiated by Japan.

If one learns from the history of previous epidemics and pandemics, one can expect changes in global health this time too. Furthermore, they will be different from previous ones. The COVID-19 pandemic affected wealthy nations first, and the magnitude is substantially larger in geographic area and scale. These have resulted in a situation that no one could help other. For example, the health systems in Italy were suddenly overwhelmed by a sharp rise in critical cases [7]. At that time, all G7 nations are struggling at their home counties, and even neighboring France and Germany could not afford to help Italy. Everyone recognized quickly that the only way to save oneself is to accelerate R&D for new vaccines, therapeutics, and diagnostics, and to ensure accessibility by mass production with appropriate regulatory and pricing controls for everyone, rich or poor, on our planet. Also, the time slot to do so is very short to prepare for the next wave of outbreaks and the next influenza season. This is very distinct departure from the traditional concept of international health, which in principle is based on rich countries helping poor countries by supplying financial and other resources, using official development aids (ODA). We will see an exciting evolution of global health in the next 12 months. Still, the focus will be placed on R&D in developing new vaccines, therapeutics, and diagnostics as well as strategies in scaling up of both production and utilization. In this paper, the authors describe the global landscape of R&D in infections with particular attention to neglected diseases. Also, we will look into the emerging mechanism to support the R&D and the associated endeavors to translate R&D into actual provision of medical and health products. Finally, we will look at possibilities of the role of new funding models, taking an example of the GHIT Fund. Scientists and researchers have long wished to help the needed. They have the chance to do so this time.

## Global landscape of R&D

The first two decades of the twenty-first century will be remembered as glorious years of progress in human history. People became wealthier and healthier. Table 1 shows the ranking of causes of death by country income category. The global top three killers are ischemic heart disease, stroke, and obstructive pulmonary disease, which may be surprising to many. However, the low-income countries are still struggling with infectious diseases, but their share of the global population is only 9.8%. The mortality ranking in these countries is very similar to that of Japan in 1950, exactly 70 years ago. These figures explain the heavy allocation of R&D resources for non-communicable diseases, the predominant health challenges in middle-income (MIC) and high-income countries (HIC) accounting for nearly 90% of the global population with the power to purchase health products. However, there is a group of diseases that predominantly affects low-income countries (LIC). For example, in view of mortality, morbidity, and disease burden, HIV, tuberculosis, and malaria disproportionally affect LIC, and are referred to as the “big three (infectious diseases)”. Furthermore, there is another group of diseases that affect the poorest people among the poor in LIC where health services and drugs are often unaffordable, inaccessible, and unavailable for those who have weak sociopolitical and socioeconomic status, leaving them “neglected”. WHO categorized these diseases as neglected tropical diseases (NTDs), which consist of 20 diseases primarily caused by pathogens such as bacteria, parasitic helminths, protozoa, fungi, viruses, and ectoparasites, as listed in Table 2.

**Table 1:** The top 10 causes of death

Ranking	2000 (World)	2016^**a**^					For reference^**b**^	
		World	High-income countries	Upper-middle income countries	Lower-middle income countries	Low-income countries	Japan (2018)	Japan (1950)
**1**	Ischemic heart disease	Ischemic heart disease	Ischemic heart disease	Ischemic heart disease	Ischemic heart disease	Lower respiratory infections	Malignant neoplasms	Tuberculosis
**2**	Stroke	Stroke	Stroke	Stroke	Stroke	Diarrheal diseases	Heart diseases	Cerebrovascular diseases
**3**	Lower respiratory infections	Chronic obstructive pulmonary disease	Alzheimer disease and other dementias	Chronic obstructive pulmonary disease	Lower respiratory infections	Ischemic heart disease	Senility	Pneumonia or bronchitis
**4**	Chronic obstructive pulmonary disease	Lower respiratory infections	Trachea, brochus, lung cancers	Trachea, brochus, lung cancers	Chronic obstructive pulmonary disease	HIV/AIDS	Cerebrovascular diseases	Gastroenteritis
**5**	Diarrheal diseases	Alzheimer disease other dementias	Chronic obstructive pulmonary disease	Alzheimer disease and other dementias	Tuberculosis	Stroke	Pneumonia	Malignant neoplasms
**6**	Tuberculosis	Trachea, brochus, lung cancers	Lower respiratory infections	Lower respiratory infections	Diarrheal diseases	Malaria	Accidents	Senility
**7**	HIV/AIDS	Diabetes mellitus	Colon and rectum cancers	Diabetes mellitus	Diabetes mellitus	Tuberculosis	Aspiration pneumonia	Heart diseases
**8**	Preterm birth complications	Road injury	Diabetes mellitus	Road injury	Preterm birth complications	Preterm birth complications	Renal failure	Other neonatal-specific diseases
**9**	Trachea, brochus, lung cancers	Diarrheal diseases	Kidney diseases	Liver cancer	Liver Cirrhosis	Birth asphyxia and birth trauma	Vascular/unspecified dementia	Accident
**10**	Road injury	Tuberculosis	Breast cancer	Stomach cancer	Road injury	Road injury	Suicide	Nephritis or nephrosis
Population^C^	6.09 billion	7.46 billion	1.24 billion	2.59 billion	2.91 billion	0.73 billion	0.126 billion	0.083 billion
Population%		100.0%	16.6%	34.7%	38.9%	9.8%	1.7%	3.3%

^a^WHO: https://www.who.int/news-room/fact-sheets/detail/the-top-10-causes-of-death

^b^Ministry of Health, Labour and Welfare: https://www.mhlw.go.jp/toukei/saikin/hw/jinkou/geppo/nengai18/dl/gaikyou30.pdf

^c^UN: https://population.un.org/wpp/DataQuery/

**Table 2:** WHO list of neglected tropical diseases (NTDs)

• Buruli ulcer	• Mycetoma, chromoblastomycosis, and other deep mycoses
• Chagas disease	
• Dengue and chikungunya	• Onchocerciasis (river blindness)
• Dracunculiasis (guinea-worm disease)	• Rabies
• Echinococcosis	• Scabies and other ectoparasites
• Foodborne trematodiases	• Schistosomiasis
• Human African trypanosomiasis (sleeping sickness)	• Soil-transmitted helminthiases
	• Snakebite envenoming
• Leishmaniasis	• Taeniasis/Cysticercosis
• Leprosy (Hansen’s disease)	• Trachoma
• Lymphatic filariasis	• Yaws (endemic treponematoses)

Source: https://www.who.int/neglected_diseases/diseases/en/

Røttingen et al. [8] attempted to identify the research gap between health R&D and disease burden. First, they categorized the diseases by the ratio of disease burden of low- and middle-income countries to high-income countries into type I (less than 3), type II (3 to 35), and type III (above 35). For example, the ratio for Chagas disease is 1869, meaning that the disease burden of Chagas diseases is 1869 times higher in the low- and middle-income countries than in high-income countries. Most NTDs and malaria belong to type III, while HIV and tuberculosis belong to type II. These diseases have often been ignored by the pharmaceutical industry due to the lack of commercial incentives, and therefore, research budget is comparatively limited. Hence, the big three and NTDs combined are often referred to as “neglected diseases,” while recently antimicrobial resistance (AMR) is also widely recognized as another area of under-investment. In the scientific community, coronavirus was hardly recognized prior to the outbreak. If one searches the literature in PubMed using the keyword “coronavirus” from 2015 to 2019, the total number of publications is 3915, which is about 5% of the number of publications relating to HIV (76,493) and 0.4% of publications relating to cancer (932,959) during the same period.

Why is there such a vast difference in research based on demand and supply? From the demand perspective, the size of the affected population and disease severity are prerequisite factors for priority setting in research. Also, from the perspective of industry, whether a society can afford to purchase drugs or other health products has a great impact on R&D efforts. By ensuring adequate resources for purchase, the gap between health needs and demands will be closer. HIV/AIDS started with insufficient demand. The disease was first reported as a rare disease specific to a particular population, but the epidemic evolved into a national crisis in Africa in 2000, where most of the countries were poor and had little purchasing power. The G7-led global response began at the Kyushu-Okinawa Summit in 2000, which resulted in the creation of the Global Fund to fight AIDS, Tuberculosis, and Malaria, an international financing and partnership organization. The Global Fund now provides 4 billion USD annually to help developing countries to purchase drugs, other commodities, and services. In response to ample resource for purchase, the production of medications and related R&D expanded, and the supply caught up with the demand. The flow of ODA is highlighted by the Financing Global Health Report published by the Institute of Health Matrix and Evaluation [9]. The health ODA in 2019 was 41 billion USD, and 23% or 9.5 billion USD was allocated to HIV, while other infections such as Ebola and other non-NTDs received only 6% or 2.3 billion USD.

Research funding is another important topic. It is highly challenging to collect all the information from both public, private for-profit, and non-profit sectors. Røttingen et al. estimated that the total research resources amounted to 214 billion USD in 2009, of which 60% came from the industry, 30% from governments, and 10% from philanthropy. The allocation to NTDs was 1%. In 2018, health R&D investment in the pharmaceutical industry was 179 billion USD [10]. If the share of industry remains at 60%, the total R&D resources would be 298.3 billion USD. This underfunded situation to address unique health challenges alarmed the World Health Organization (WHO), which then created a dedicated working group called “Consultative Expert Working Group on Research and Development: Financing and Coordination” and prepared a report [11].

Their recommendation included numerical targets for research budget dedicated to the relevant research in developing countries that failed to obtain sufficient support from donor countries. Some of the recommendations, including the creation of a dashboard of R&D information, were materialized as the Global Observatory on Health R&D [12], which includes the R&D funding flow for neglected diseases (G-FINDER) [13]. The G-FINDER 2019 collected funding information from 262 private, public, and philanthropic organizations on all types of product-related R&D, basic research, and platform technologies covering 36 neglected diseases in 2018. The total amount of funding was approximately 4 billion USD, 62% of which came from governments, 19% from foundations, 17% from industry, and 2% from other sources. Japan contributed 33 million or 0.9% of the total amount. However, this amount did not include R&D grants from the Japan Agency for Medical Research and Development (AMED) [14] the principal health funding agency of Japan, which funds 54 research projects on emerging and re-emerging diseases, and recently added five grants for COVID-19 research. Concerning target diseases of G-FINDER 2019, “neglected diseases” encompass the above-mentioned “big three” and NTDs. Out of the 4 billion USD, 70% went to HIV, TB, and malaria, and the remaining 30% to other infectious diseases including hepatitis C and bacterial pneumonia. Hence, resources dedicated to NTD are calculated to be as low as 834 million USD: 8% (83 million USD) from the industry, 24% (227 million USD) from philanthropy, and 68% (532 million USD) from public. Therefore, the share of R&D resources for NTD provided by the industry actually amounts to around 0.05% (0.083 billion/179 × 100). This highlights the small appetite for R&D in NTD shown by the industry, due to non-existing commercial incentive or unpredictable demand for health products. Further examination of funding distribution confirms this situation: 41% goes to academia, 22% to industry, 14% to partnership, and 12% to public research institutions. By area of investment, 30% is for vaccine, 26% for drugs, 5% for diagnostics, and 39% to others. The pitfalls of this funding situation become visible in the COVD-19 pandemic. Due to poor research funding and lack of interest from the industry, R&D on diagnostics receives the lowest amount of funding. This situation may have contributed partially to the delay in large-scale production of validated high-quality diagnostics after the disease emerged.

## Endeavor to enhance access

COVID-19 is caused by the SARS-CoV-2 virus, a new coronavirus transmitted to humans sharing similarities of previous coronaviruses causing severe acute respiratory syndrome (SARS) and *Middle East respiratory syndrome* (*MERS*). Despite the concern expressed by health security communities [15], coronavirus infections remained under-funded and consequently under-studied, due to relatively limited global health impact by SARS and MERS. Hence, no rapid diagnostics, treatments, and vaccines exist. The world needs to be united to develop the necessary health technology, and eventually to organize mass production and distribution according to the need of nations and their populations. However, the reality may be far from ideal. Those nations that are successful in developing new technology will enjoy benefit first, and other countries may have access to the technology later and often at a higher price. Unfortunately, the global geopolitics is seen as an exacerbation factor if one considers the call for an impartial, independent, and comprehensive evaluation of WHO demanded by the World Health Assembly Resolution [16] and the clear strategic motive of the two most significant economic powers in the world. The two countries are not shy about treating essential technologies as strategic tools. Under such circumstances, the world is trying to mitigate the risks of not having access to essential technology and products.

Table 3 illustrates the various steps toward access of global products, from recognizing the emergence of infection to delivery of health products to the needed populations. The R&D plays critical roles, but without other elements, the research outcomes cannot help solve health problems on a massive scale. Needless to say, the operational and policy research also plays important roles to ensure smooth implementation in the field and to formulate policies. In each step, the existing mechanisms and actors differ. Let us examine them one by one.

**Table 3:**
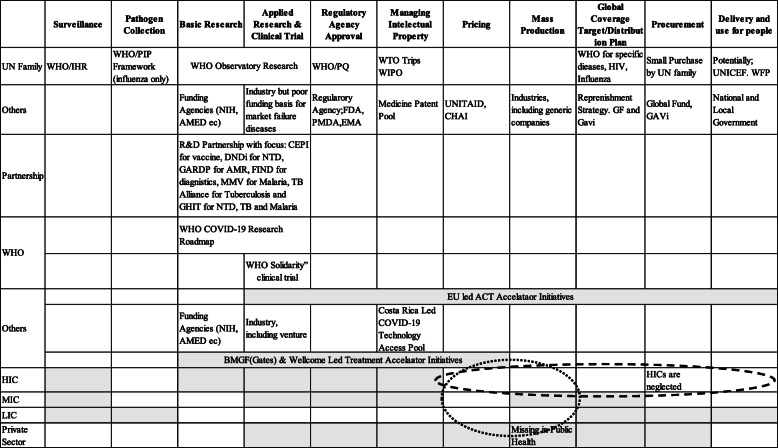
Steps towards access to global goods

### Step 1: surveillance

Recognition of the emergence of infections is imperative. According to the International Health Regulations (IHR 2005) [3], WHO member states agreed to work together to build their capacities to detect, assess, and report public health events. Using this mechanism, the world can know what infection is emerging where. Then, WHO should advise and alert countries, for example, by issuing Public Health Emergency of International Concern (PHEIC) [17]. This step was exercised in the case of COVID-19, but whether WHO response was adequate and timely is being questioned by several countries. The Global Virome Project was launched in 2018 aiming to predict pathogens that could produce lethal pandemics [18].

### Step 2: pathogen collection

To start research, the pathogen needs to be provided by the affected countries. Even in the event of H1N1 influenza pandemic in 2009, some countries were still reluctant to share virus strains unless they were comfortable that the vaccine produced was provided at affordable prices. To address such concerns of developing countries, the Pandemic Influenza Preparedness (PIP) Framework [4] was agreed by all WHO member states in 2011 to enhance smooth collection of influenza virus and access to health products developed. Since China shared the COVID-19 gene sequence at an early stage, and outbreaks occur in multiple countries, the provision of pathogen was not an issue in the case of COVID-19.

### Step 3: basic research; step 4: applied research and clinical trial

WHO facilitates information sharing through the WHO Observatory on Health R&D [12], but the actual provision of financial resources comes from various entities, as discussed earlier. In recent years, product development partnerships are emerging, which stimulate relevant research in various domains. Examples include CEPI [5] for new emerging infections such as Ebola, GHIT fund [5] and DNDi [19] for NTDs, Global Antibiotic Research and Development Partnership (GARDP) [20] for drug-resistant bacteria, FIND [21] for diagnostics, MMV [22] for malaria, and TB Alliance for tuberculosis [23]. However, these partnerships have rather narrow mandates. Hence, there is room for new initiatives specifically targeting COVID-19. With this background, a group of international experts published the WHO Coordinated Global Research Roadmap in March 2020 [24] which lists priority research to accelerate R&D directly relevant to COVID-19. In addition, to facilitate sharing of outcome of clinical trials to avoid duplications, dedicated cyberspace entitled WHO Solidarity Clinical Trial for COVID-19 Treatment [25] is available.

In the international arena, international partnerships have been formed. For example, the Bill and Melinda Gates Foundation of the USA and Wellcome Trust of the UK joined forces to boost R&D trough COVID-19 Therapeutics Accelerator [26] in March 2020, and EU launched the Global Collaboration to Accelerate the Development, Production and Equitable Access to New COVID-19 Diagnostics, Therapeutics, and Vaccines, in short, Access to COVID-19 Tools (act) Accelerator [27] in April 2020. The uniqueness of these initiatives is that their focus is not limited to R&D but covers the subsequent steps to lead production and mass application.

### Step 5: regulatory agency approval

The products produced need careful evaluation of safety and efficacy by regulatory agencies such as Pharmaceuticals and Medical Devices Agency (PMDA) in Japan, Food and Drug Administration (FDA) in the USA, and European Medicines Agency (EMA) in EU countries. These three agencies are highly respected as the “stringent regulatory authority”. Also, WHO has a regulatory mechanism called prequalification (PQ) [28] to assess generic products to maintain the minimum standard for international purchase. Products need approval from stringent regulatory agencies or WHO PQ, which by itself is a challenging step for moving ahead quickly.

### Step 6: managing intellectual property

Developers of innovative health products naturally wish to demand protection of intellectual property (IP) right, the general rules of which are governed by the World Intellectual Property Organization (WIPO). Another side of the coin is that IP is an obstacle for technology transfer to other manufacturers, which may be a serious issue when mass production is required. An arrangement was made under the Agreement on Trade-Related Aspects of Intellectual Property Rights (TRIPS) [29] established by the World Trade Organization (WTO), which sets minimum standards for the handling of IP issues by national governments in health products under health emergencies. However, this mechanism has been regarded not as effective as initially thought, and Medicines Patent Pool [30] was created to serve as a one-stop-shop for handling IP in the health sector. In May 2020, Costa Rica initiated the creation of COVID-19 Technology Access Pool [31] which covers broader IP issues beyond those related to health products. It additionally includes genetic information, clinical trials, contract information, and supplementary know-hows for technology transfer.

### Step 7: pricing

The manufacturers consider the price before expanding production capacity. Pricing is decided by the corporate body of each company. However, for those global goods, one may ask whether it is appropriate to leave this critical step entirely in the hands of the industry. Hope to influence pricing through market dynamism, UNITAID [32], which was established by like-minded countries that agreed to contribute their air ticket levies, and Clinton Health Access Initiatives (CHAI) [33] have been working to reduce the prices of medicines for HIV, TB, malaria, and hepatitis, and now move to cancer and other areas.

### Step 8: mass production

This step is generally the responsibility of the industry, and not enough attention has been paid to this aspect by global health community. However, the EU initiative, Access to COVID-19 Tools (ACT) Accelerator [27], is looking at the possibility to offer cross-border support to manufacturers in other nations for mass production of “global goods.”

### Step 9: global coverage target and distribution plan

For the manufacturers’ point of view, they cannot expand capacity without knowing the volume of products to be produced and how they will be purchased and distributed. Traditionally, WHO develops a global plan. For example, in the past, WHO set the target to treat 15 million HIV patients in sub-Saharan African countries. In supporting such target, Global Fund organized replenishment to ensure sufficient funds to realize the target [34]. In the case of vaccines, the Global Alliance for Vaccines and Immunization (Gavi) [35] adopts a similar approach.

### Step 10: procurement

UN families procure health products, but the volume is not too large. On the other hand, Global Fund and Gavi provide supports for low-income countries.

### Step 11: delivery and use

This is primarily decided by each country. The role of international organizations is helping countries in crisis. The central teams set global norms and standards such as treatment guidelines, and country teams of WHO primarily offer technical advices.

In Table 3, the beneficiaries are shown at the bottom of each step. Note that the organizations and mechanisms listed in Table 3 are illustrative and simplified examples, which reflect a subset of players in each step. Also, the organizations involved are more diverse and steps may not be clearly demarcated.

## New funding model and way forward

Many infectious diseases from the past are under control, and even smallpox has been eradicated. However, new infectious diseases are emerging, antimicrobial resistant bacteria are increasing, and the interval of their assault on human society is shortening. Global communities have made efforts to help low-income countries where many vulnerable populations live. It is reasonable to invest more in developing countries to help stop the vicious cycle of infectious diseases and poverty. Plenty of success cases were witnessed in the first two decades of the twenty-first century. But, this time around, COVID-19 started in the world’s second-largest economy, and then spread to high-income countries or regions such as Europe and the USA before moving to other BRICs countries, Latin America and Africa. None of the developed countries could help each other as they had their hands full at home. In many countries, health systems were overwhelmed, and the scene was akin to the fragile medical situation seen in developing countries every day. The same applied to health resources. Severe shortage of health personnel, ventilators, and even personal protective gear seemed like another daily scene in developing countries. The middle-income countries are facing even harder reality because the numbers of infection are still rising. This reminds the leaders of these countries the real responsibility of graduation from being funded by overseas assistance toward funding domestically to provide sustainable preventive and curative services for their own nationals, which account for 73.6% of the global population (Table 1). These situations urge global health communities to rethink the essence of global health: Is it charity or rather shared responsibility of all human beings?

Perhaps, this perception of paradigm shift is one of the positive impact of the COVID-19 pandemic. As shown in Table 3, the missing area of beneficiary is developed countries. In other words, there is no existing mechanism that specifically benefits developed countries. Let us look ahead to the world when vaccines are successfully developed. The present arrangements have attracted resources from many countries including Japan. Is it rational if the vaccines go to developing countries, but not Japanese nationals? It is reported that Prime Minister Abe proposed joint patent management for mass production. Another idea is to develop a pool of resources to make advanced purchase commitments of health products for everyone, regardless of the wealth status of countries. Populations not targeted by the current mechanisms of supply of medicines and vaccines, i.e., nationals of high-income countries, as well as various steps leading to access will be the focus of ongoing discussions. Many R&D mechanisms, including the GHIT Fund, realize that without linkage to access to the novel health technologies and products generated, the mission of helping people affected by NTD, COVID-19, or antimicrobial resistance is not complete. At the same time, discussion of access is meaningless until we have persuasive products at hand. Therefore, we strongly believe that the R&D and access are inextricably connected.

In the absence of a leading global power, there is a potential opportunity for Japan to serve as a consensus builder as it did in the Kyushu-Okinawa summit in 2000 (creation of Global Fund), in the Toyako summit in 2008 (strengthening health system), and in the Iseshima summit in 2016 (universal health coverage). In this context, the GHIT Fund also can serve as a useful model for the R&D of innovative health products. The Japanese-based GHIT Fund is an international public-private partnership for global health R&D. It is founded by the Japanese Ministry of Health, Labour and Welfare; Ministry of Foreign Affairs; international philanthropy organizations (Bill & Melinda Gates Foundation and Wellcome Trust); and the Japanese pharmaceutical and diagnostic industry on a share basis of 25% each. The GHIT Fund leverages the Japanese industry, academia, and research institutes, in collaboration with global partners to create new drugs, vaccines, and diagnostics for neglected tropical diseases. The lack of market incentives has resulted in the neglect of its R&D. In our view, our model brings four direct benefits. First, by the GHIT funding model, we can offer a strong stimulus for the industry. They can expect high public recognition by participating in international projects in alleviating the misery of the poorest among the poor through their core business of R&D. Also, they can gain access to new research funds, including GHIT’s investment, and further research partners. Second, the Japanese Government expects merit of global recognition and enhancement of their contribution from essential players of R&D, i.e., industry and philosophy organizations. Third, international philanthropy organizations rest assured of robust government-backed oversight. Lastly, all parties should be happy about a balanced position of the Fund, based on its governance structure involving all stakeholders and mobilization of each party’s strengths. Beginning the seventh year since inception, GHIT aims to deliver two products to low-income countries to alleviate the burden of neglected diseases: a rapid diagnostic tool for tuberculosis and an improved formulation of praziquantel for schistosomiasis. Therefore, we believe that the GHIT Fund model is unique and useful for targeting the R&D of market-failure products by offering the abovementioned quadruple wins for all partners. The recent establishment of R&D fund with similar concept, such as CEPI (Coalition for Epidemic Preparedness Innovations) and the Right Fund (Research Investment for Global Health Technology Fund) [36], supports our argument.

Many agree that we cannot return to the old days after the COVID-19 pandemic. Health R&D will not be exempted. R&D specialists need to be aware of the change in the R&D environment and adjust accordingly. If we can link our endeavors to the global sequences of actions, we can surely benefit everyone, including our fellow nationals.

## Data Availability

Not applicable
